# Enhancing Hemoglobin Levels in Moderately Acute Malnourished Children Aged 6–59 Months: A Randomized Controlled Trial of a Novel Ready‐to‐Use Food (RUF)

**DOI:** 10.1155/anem/8863009

**Published:** 2025-12-28

**Authors:** Nyabasi Makori, Ray Masumo, Suleiman Rashid, Theresia Jumbe, Meshack Tegeye, Debora Esau, Juliana Muiruri, Geofrey Mchau, Cypriana Moshi, Neema Shosho, Vera Lugutuah Kwara, Hoyce Mshida, Stanslaus Henry, Elizabeth Lyimo, Germana Leyna, Hope Masanja

**Affiliations:** ^1^ Department of Nutrition Education and Training, Tanzania Food and Nutrition Centre, P.O. Box 977, Dar es Salaam, Tanzania; ^2^ Department of Community Health and Nutrition, Tanzania Food and Nutrition Centre, P.O. Box 977, Dar es Salaam, Tanzania; ^3^ Department of Food Science and Agroprocessing, Sokoine University of Agriculture, P.O. Box 3000, Morogoro, Tanzania, suanet.ac.tz; ^4^ 4, Department of Human Nutrition and Consumer Sciences, Sokoine University of Agriculture, P.O. Box 3006, Morogoro, Tanzania, suanet.ac.tz; ^5^ World Food Programme, P.O. Box 77778, Dar es Salaam, Tanzania; ^6^ Department of Food Science and Nutrition, Tanzania Food and Nutrition Centre, P.O. Box 977, Dar es Salaam, Tanzania; ^7^ Department of Epidemiology and Biostatistics, Muhimbili University of Health and Allied Sciences, Dar es Salaam, Tanzania, muchs.ac.tz

**Keywords:** hemoglobin concentration and nutritional intervention, iron deficiency anemia, moderate acute malnutrition (MAM), ready-to-use food (RUF)

## Abstract

Iron deficiency anemia among children with moderate acute malnutrition (MAM) presents a significant challenge that can negatively impact treatment outcomes. This burden and its contributing factors among children with MAM in Tanzania prompted the current study, which aimed to assess the efficacy of ready‐to‐use food (RUF) supplements in increasing hemoglobin levels specifically among children with MAM aged 6–59 months. A total of 271 children (RUF: 91; CSB+: 90; standard of care: 90) recruited for the study, and the randomized controlled trial employed a three‐parallel‐arm design; the first arm received corn soy blend plus (CSB+) with infant and young child feeding (IYCF) counseling, the second arm received RUF and IYCF counseling, and the third arm served as a control group. The intervention was administered over 3 months, following the WHO guidelines for intervention studies. Results indicated a mean increase of 2.70 g/dL in mean hemoglobin (Hb) concentration (at *p* ≤ 0.01) among the RUF intervention arm. Similarly, the CSB arm showed an increase in mean Hb concentration from 9.88 g/dL to 11.88 g/dL (*p* ≤ 0.01). In contrast, the standard of care arm experienced a decrease in mean Hb levels by 0.25 g/dL (*p*  >  0.05) at the end line. Additionally, the prevalence of anemia was significantly reduced from 65.90% to 27.20% at baseline and at the end of the study, respectively, with a notably lower prevalence of 14.3% in the RUF arm as compared to the standard of care arm of 44%. The study provides strong evidence that RUF is efficacious in improving Hb concentration, a key biomarker for nutritional anemia among children with MAM, and successfully reduces the prevalence of anemia.

## 1. Introduction

Moderate acute malnutrition (MAM) is a form of malnutrition that results from a sudden reduction in food intake or diet quality, often with pathological causes in children. The condition is highly prevalent in low‐income countries [1]. As MAM is associated with poor dietary quality, moderate malnourished children are also likely to suffer from anemia. According to the World Health Organization (WHO) estimates, anemia is a serious public health problem. Globally, the burden of anemia is highest among pregnant women (42%), followed by children under 5 years (40%), making it a severe public health concern. Sub‐Saharan African countries have a high burden of anemia especially in regions with lower socioeconomic development [2]. The disease is responsible for 58.6 (40.14–81.1) million years lived with disability [3]. Although the etiology of anemia is multifactorial, approximately 50% of its prevalence is attributed to iron deficiency (ID) [4, 5]. Anemia is defined as having hemoglobin (Hb) levels below a certain cutoff point depending on the age of the population being evaluated, as well as physiological status, gender, and altitude [6]. Hb is the main oxygen carrier in humans; when there are too few red blood cells, or when they are abnormally shaped, the blood’s ability to carry oxygen to the body’s tissues is reduced [7]. During childhood, maintaining an optimal Hb concentration is crucial for a child’s physiologic needs; failure to do so can lead to anemia. Consequently, anemia can hinder cognitive and motor development in children and contribute to stunting.

Prevention of micronutrient deficiencies is critically important. The Tanzanian government is implementing the National Multisectoral Action Plan II 2021/26 to address malnutrition including micronutrient deficiencies. Efforts to combat the deficiencies have included food‐based approaches to enhance diet diversity, fortification of foods, biofortification, supplementation, and social and behavior change communication [8]. For children under 5 years of age, preventing anemia primarily involves promoting the consumption of diversified foods, optimal breastfeeding, the intake of iron‐rich foods, and the use of multiple micronutrient powders. Despite these efforts, the burden of anemia among this group remains high, at 47.10% according to the TDHS 2022, and only 19% of children reported consuming diversified foods [9].

In 2022, the government of Tanzania with support from academia and development partners developed a locally accessible, nutrient‐dense ready‐to‐use food (RUF) that has been demonstrated to be efficacious in the dietary management of MAM. Considering the co‐occurrence of malnutrition and anemia, it is crucial for the food product used for the dietary management of MAM children to be efficacious in treating both anemia and MAM. In Tanzania, locally produced RUF has been proven to be efficacious in managing MAM [10]. The RUF product formulation comprised maize, millet, soybean, sorghum, sesame seeds, and sugar. The product was minimally fortified with a micronutrient premix that included iron, zinc, vitamin B_12_, and folic acid. To establish the relative effect, the study compared the locally produced RUF with the conventionally used corn soy blend plus (CSB+), which is also fortified and widely used in supplementary feeding programs. The RUF had slightly higher energy density and fat content, whereas both products contained similar levels of iron and other key micronutrients. The primary hypothesis was to test whether children receiving locally produced RUF would show a greater improvement in Hb concentration compared to those receiving CSB+ or infant and young child feeding (IYCF) counseling alone.

Findings from other studies have reported that RUF is efficacious in treating moderate wasting (weight gain) and micronutrient deficiencies in children aged between 6 and 24 months [11]. However, further evidence is needed to evaluate the efficacy of locally produced ready‐to‐use supplementary food at the community level for addressing anemia in Tanzania. Therefore, this study aimed to determine the effect of providing 100 g of RUF daily for 3 months versus the conventional CSB+, on Hb concentration among children aged 6–59 months in Tanzania. The findings from this study will provide evidence to the Tanzanian government regarding the efficacy of locally developed food supplement (RUF) in addressing anemia and improving the nutritional status of children aged 6–59 months in Tanzania.

## 2. Materials and Methods

### 2.1. Study Setting

The study was conducted from October to December 2023 in the Dodoma and Singida regions, specifically in the Chamwino and Ikungi districts. The regions, located in the central zone of Tanzania, are characterized by high food insecurity and child malnutrition. The districts were purposely selected because they are beneficiaries of food aid, specifically CSB+ flour from the World Food Programme (WFP). The Hb recovery trial took place in six (6) selected health facilities (three health facilities from each district, Chamwino and Ikungi). In each district, a selected health facility represented one of the intervention arms: RUF, CSB, and standard of care arms.

### 2.2. Sample Size and Study Participants

A subsample of 50% of the total participants from the larger cohort described in [10] was included in a mini‐study for Hb assessment. Consequently, a total of 247 mother–child pairs with children with MAM aged 6–59 months were recruited as study subjects.

### 2.3. Sampling and Recruitment of Study Participants

Villages were randomly selected from each district, one health facility from each selected village was included, and recruitment of study participants was performed at the health facility level after screening for eligibility. In each district, three villages were randomly selected and assigned to one of the three intervention arms (CSB, RUF, or standard) using simple random sampling. Participants who met the inclusion criteria were randomized using random numbers, based on the recruitment targets for each health facility. The inclusion criteria consisted of a weight‐for‐height z‐score (WHZ) less than −2, midupper arm circumference (MUAC) less than 12.5 cm, age of a child between 6 and 59 months, residency in the catchment area, a signed informed consent form, no current intake of RUFs, and the availability of the mother–child pair for the next three months. Recruited children were identified as having MAM based on their MUAC and weight‐for‐height/length z‐scores.

### 2.4. Study Design and Intervention

This study was an open‐label, randomized controlled trial assessing the efficacy of 100 g/day of RUF compared with 100 g/day of CSB+ in increasing Hb concentration among children with MAM over a period of three months (12 weeks). The trial included three parallel arms: The first arm served as the intervention group, which received CSB+ along with routine IYCF counseling from health professionals. The second arm, similar to the first, received RUF and IYCF counseling. The third arm was the control arm, which received IYCF counseling only, without any supplementation. In all three arms, intervention was administered for 3 months, in accordance with the recommended duration set by the WHO for an intervention study. Mothers and caregivers of children in the RUF and CSB+ arms were provided with specific recommendations on how to use the products based on the type assigned.

### 2.5. Intervention Administration and Blinding

Children in the intervention arms (CSB+ and RUF) received a daily ration of 100 g of flour for 12 weeks. Distribution of RUF and CSB+ flour was scheduled every two weeks; mothers/caregivers received a package of 14 packs, sufficient for 14 days (one pack for a day). Before distribution, caregivers attended a 10–20‐min session where they were reminded about the correct usage of RUF and CSB+ products, including preparation and cooking methods. To monitor consumption, caregivers were asked to return empty RUF and CSB+ packets at the next visit. Mothers/caregivers recruited in the standard of care arm attended IYCF counseling sessions. The content of the sessions was developed based on national guidelines and validated educational materials, aimed at equipping mothers with the knowledge necessary for optimal infant feeding to promote child growth. Counseling sessions were scheduled every 2 weeks to facilitate the required dietary changes throughout and beyond the intervention period. The study was open‐label, meaning participants were aware of the main type of food they were receiving (RUFs or CSB+). However, within the CSB+ and RUF arms, participants were blinded to the specific content of the food.

### 2.6. Data Collection and Follow‐Up

Pilot testing was conducted on the questionnaires, which were refined for clarity and accuracy. Interviews were carried out with mothers/caregivers of children aged 6–59 months who had MAM. The child’s weight was measured using a Seca weight scale (HD‐386‐BK; Tanita, Tokyo, Japan), accurate to the nearest 0.1 kg. Data collectors ensured the scale was positioned on a flat, firm surface, and weighing was performed with the child in no or light clothing. The weight of each child was measured every 2 weeks using the same equipment. Recumbent length of a child was measured using a length board (210; Seca, Hamburg, Germany) to the nearest 0.1 cm.

Trained enumerators conducted face‐to‐face interviews using a pretested semistructured​ questionnaire administered using Survey Solution software version 21.09.58 [12]. Data were collected in seven phases throughout the study period. Baseline and end‐line questionnaires were administered once at the beginning and end of the study, respectively. The interviews aimed to gather information on various aspects from recruited mother–child pairs, including sociodemographic and household information, antenatal care, infant health, health‐seeking behavior, infant feeding practices, household WASH facilities, exposure to educational sessions, and household food security.

Minimum dietary diversity (MDD) was estimated by using dietary information collected from a 24‐h dietary recall. The dietary diversity of the studied population was measured using a scale of seven food groups as per the Food and Agriculture Organization (FAO) to measure dietary diversity scores (DDSs) [13]. The assessment was based on the number of food groups consumed in the past 24 h. The food groups included cereals, roots and tubers, legumes and nuts, fruits and vegetables, eggs, dairy products, and vitamin A–rich food. Each food group consumed over the reference period received 1 point, and the DDS was calculated as the sum of all points scored. A respondent with a total score of less than 4 points did not meet MDD. To assess the consumption and adherence of porridge feeding in both the RUF and CSB+ arms, a porridge feeding questionnaire was administered every 2 weeks (five times in total). During the visits, caregivers were interviewed to report daily consumption, and this approach allowed the study team to monitor compliance with the recommended daily ration and provide timely counseling to caregivers when deviations were observed.

Hb concentration was measured using a blood sample collected by pricking a finger. The finger was wiped with an alcohol‐soaked cotton swab for hygiene and safety measures. The pricked finger was gently pressed to allow the flow of blood, and a blood sample was placed in a microcuvette, which was then inserted into the HemoCue photometer machine (EFK Diagnostics, GmbH, Germany). The Hb level was read and recorded immediately. Anemia was determined based on the WHO guidelines, and categorization of Hb level was carried out as per the WHO reference standards [14].

Hb level was measured twice, at the baseline and at the end of the study, to assess the effect of the intervention on Hb increase between arms. Hb levels < 10.5 and < 11.0 g/dL were used to characterize anemia in children aged 6–23 months and 24–59 months, respectively [14]. Additionally, Hb levels of < 7.0 g/dL were considered severe anemia.

### 2.7. Data Analysis

Data were analyzed using Stata version 18.0 (StataCorp, College Station, TX, USA). Comparisons among the three study arms were assessed using the difference‐in‐difference (DiD) method with a significance level set at *p*  <  0.05. Differences in Hb levels between the baseline and end‐line surveys were compared across groups of categorical variables. The DiD analysis is a useful method for estimating the effects of quasi; however, it can also be appropriate in RCTs to isolate the intervention’s impact from (i) consistent differences between the control and intervention groups and (ii) time‐related changes in the outcome that are not caused by the intervention [15]. Anemia prevalence was analyzed by comparing proportions between study arms at baseline and end line. Chi‐square tests were used to assess statistical differences between groups, and corresponding *p*‐values were reported. Chi‐square tests were used to assess statistical differences between groups, and corresponding *p*‐values were generated.

### 2.8. Ethics Considerations

Ethical approval for conducting this study was obtained from the National Health Research Ethics Sub‐Committee of the National Institute of Medical Research of Tanzania (NIMR/HQ/R.8a/Vol.IX/360). Permission to conduct research in the Ikungi and Chamwino districts was sought from the President’s Office, Regional Administration and Local Government. The purpose of the study, methods of data collection, confidentiality, and voluntary participation were clearly explained to mothers/caregivers of children invited to sign an informed consent form. Written informed consent was obtained from all mothers and caregivers of children who met the inclusion criteria before the recruitment. All interviews and intervention procedures were conducted in private. Children who developed SAM during the intervention period were referred to health facilities for further treatment. Those who did not recover from MAM during the trial were also referred to health facilities. Additionally, children with Hb levels < 110 g/L at the end of the intervention period were referred for further evaluation and treatment.

## 3. Results

### 3.1. The Enrollment Process and Trial Profile for the Study Participants

**Figure 1:**
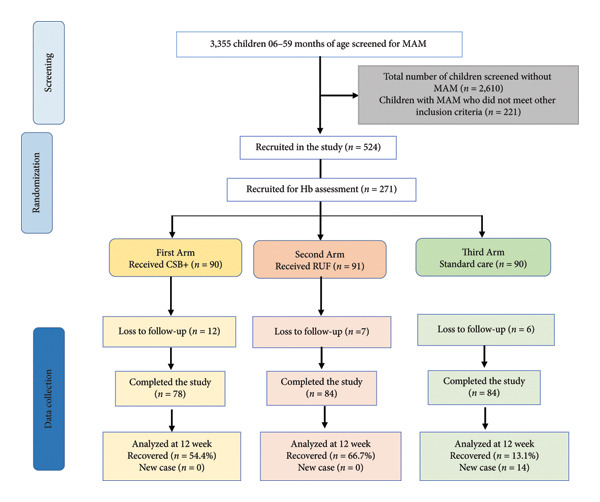
Flowchart of subjects’ enrollment and conduction of the randomized controlled trial (RCT); MAM, moderate acute malnutrition; Hb, hemoglobin; CSB+, corn soya blend plus; RUF, ready‐to‐use food.

Assessment of the eligibility of 3355 children was conducted through screening, of which 524 met the inclusion criteria. A subsample of eligible children (271 participants) was randomly selected to evaluate the effect of the intervention on increasing Hb levels across three arms: the CSB+ arm (*n* = 90), the RUF arm (*n* = 91), and the standard of care arm (*n* = 90) (Figure 1). A total of 246 (91%) recruited mother–child pairs completed the study, with 25 lost to follow‐up. The reported reasons for loss to follow‐up included moving outside the study area (due to divorce, holidays, and livelihood activities) and voluntary withdrawal by the mother from the study.

### 3.2. Sociodemographic Characteristics

Table 1 presents characteristics of mother–child pairs in each arm at baseline. A total of 246 mother–child pairs participated in the study until the end, with a mean age of 31.5 years for mothers/caregivers and 20.6 months for children among the recruited children; 46.30% were male, and 53.70% were female. More than half of the mothers/caregivers (66.30%) had primary education, whereas 91.90% were employed, either formally employed or self‐employed. Only 12.20% of mothers/caregivers had more than six children, and the majority were married/cohabiting (87.00%). The proportion of caregivers who were not the biological mother was small (9.30%), with the remainder being the biological mothers of the enrolled children. Of the sampled children, 57.7% were still being breastfed at baseline, and 44.70%, 38.20%, and 52.00% reported experiencing fever, diarrhea, and cough, respectively, within the 2 weeks before the survey. From the 24‐h dietary recall, the majority of the children (89.00%) did not meet the MDD. Differences in caregivers’ education, employment status, marital status, and MDD were observed across the study arms (*p* < 0.05), which may influence the results of the primary outcome (Hb concentration).

**Table 1:** Baseline characteristics of mother–child pairs in each arm.

	Intervention arms				
	Total *n* (%)	Standard of care *n* (%)	CSB *n* (%)	RUF *n* (%)	*p*‐value
Sex of a child					
Male	114 (46.3)	36 (42.9)	41 (52.6)	37 (44.0)	0.406
Female	132 (53.7)	48 (57.1)	37 (47.4)	47 (56.0)	
Caregiver education					
No formal education	61 (24.8)	28 (33.3)	10 (12.8)	23 (27.4)	0.023^∗∗^
Primary education	163 (66.3)	51 (60.7)	61 (78.2)	51 (60.7)	
Secondary/higher	22 (8.9)	5 (6.0)	7 (9.0)	10 (11.9)	
Employment status					
Unemployed	20 (8.1)	12 (14.3)	4 (5.1)	4 (4.8)	0.039^∗∗^
Employed	226 (91.9)	72 (85.7)	74 (94.9)	80 (95.2)	
Number of children					
1–3	108 (43.9)	32 (38.1)	36 (46.2)	40 (47.6)	0.695
4–6	108 (43.9)	41 (48.8)	34 (43.6)	33 (39.3)	
7+	30 (12.2)	11 (13.1)	8 (10.3)	11 (13.1)	
Marital status					
Single parent	32 (13.0)	15 (17.9)	3 (3.8)	14 (16.7)	0.014^∗∗^
Married/cohabit	214 (87.0)	69 (82.1)	75 (96.2)	70 (83.3)	
Primary caregiver					
Biological mother	223 (90.7)	75 (89.3)	73 (93.6)	75 (89.3)	0.559
Other	23 (9.3)	9 (10.7)	5 (6.4)	9 (10.7)	
Still breastfeed					
Yes	142 (57.7)	42 (50.0)	49 (62.8)	51 (60.7)	0.203
No	104 (42.3)	42 (50.0)	29 (37.2)	33 (39.3)	
Had a fever in the last 2 weeks					
Yes	110 (44.7)	42 (50)	37 (47.4)	31 (36.9)	0.196
No	136 (55.3)	42 (50)	41 (52.6)	53 (63.1)	
Had diarrhea in the last 2 weeks					
Yes	94 (38.2)	28 (33.3)	27 (34.6)	39 (46.4)	0.159
No	152 (61.8)	56 (66.7)	51 (65.4)	45 (53.6)	
Had an illness with a cough in the last 2 weeks					
Yes	128 (52.0)	46 (54.8)	39 (50.0)	43 (51.2)	0.817
No	118 (48.0)	38 (45.2)	39 (50.0)	41 (48.8)	
Minimum dietary diversity (MDD)					
No	219 (89.0)	83 (98.8)	59 (75.6)	77 (91.7)	< 0.001^∗∗^
Yes	27 (11.0)	1 (1.2)	19 (24.4)	7 (8.3)	
	Mean (SD)	Mean (SD)	Mean (SD)	Mean (SD)	
Age of caregiver (years)	31.5 (9.2)	31.8 (10.0)	32.1 (9.2)	30.5 (8.3)	0.509
Age of a child (months)	20.6 (12.7)	20.9 (13.9)	20.6 (11.6)	20.2 (12.5)	0.932

^∗∗^There is a significant difference (*p*  <  0.05).

At the end line, the mean age of children was 23.4 months, and 52.0% of all children were still being breastfed (Table 2). The proportions of children who experienced fever, diarrhea, and cough in the 2 weeks before the survey decreased compared to baseline (21.10%, 19.50%, and 20.30%, respectively). Experiencing fever and cough, as well as meeting MDD from 24‐h dietary recall, showed significant differences across study arms (*p* < 0.05).

**Table 2:** End‐line characteristic in each arm.

Variable	Total *n* (%)	Intervention arms			
		Standard of care *n* (%)	CSB *n* (%)	RUF *n* (%)	*p*‐value
Still breastfeed					
Yes	128 (52.0)	46 (54.8)	38 (48.7)	44 (52.4)	0.742
No	96 (39.0)	38 (45.2)	27 (34.6)	31 (36.9)	
Not applicable	22 (8.9)	0 (0.0)	13 (16.7)	9 (10.7)	
Had a fever in the last 2 weeks					
Yes	52 (21.1)	27 (32.1)	8 (10.3)	17 (20.2)	**0.003^∗∗^**
No	194 (78.9)	57 (67.9)	70 (89.7)	67 (79.8)	
Had diarrhea in the last 2 weeks					
Yes	48 (19.5)	13 (15.5)	22 (28.2)	13 (15.5)	0.064
No	198 (80.5)	71 (84.5)	56 (71.8)	71 (84.5)	
Had an illness with a cough in the last 2 weeks					
Yes	50 (20.3)	22 (26.2)	8 (10.3)	20 (23.8)	**0.026^∗∗^**
No	196 (79.7)	62 (73.8)	70 (89.7)	64 (76.2)	
Minimum dietary diversity (MDD)					
No	206 (83.7)	79 (94.0)	61 (78.2)	66 (78.6)	**0.007^∗∗^**
Yes	40 (16.3)	5 (6.0)	17 (21.8)	18 (21.4)	
	**Mean (SD)**	**Mean (SD)**	**Mean (SD)**	**Mean (SD)**	
Age of a child	23.4 (12.7)	23.6 (13.8)	23.5 (11.7)	23.0 (12.5)	0.942

^∗∗^There is significant difference (*p*  <  0.05).

### 3.3. Hb Concentration Before and After Interventions

**Figure 2:**
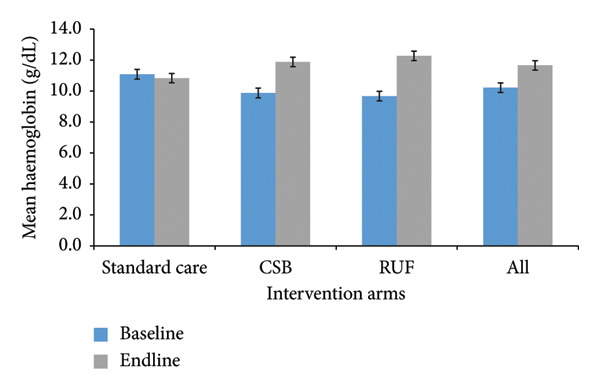
Mean hemoglobin levels (g/dL) across study arms at baseline and end line.

Figure 2 presents the changes in Hb concentration before and after the intervention period in each arm. There was a 2.70 g/dL increase in mean Hb concentration among MAM in the RUF intervention arm. Similarly, an increase in mean Hb concentration was observed among moderate acute malnourished children in the CSB+ arm, from 9.88 g/dL to 11.88 g/dL(*p* ≤ 0.01). However, there was a decrease in the mean end‐line Hb concentration compared to baseline in the standard of care arm, with a reduction of 0.25 g/dL. At baseline, 60.1% of the children were anemic, with 79.8% in the RUF arm and 40.5% in the control arm. At the end line, anemia prevalence decreased to 29.1% overall, with 14.3% in the RUF arm and 44.0% in the control arm.

Table 3 further presents the DiD analysis of the change in mean Hb concentration between the study arms. Both the RUF and CSB+ arms were efficacious in increasing the mean Hb compared to the control arm at (*p* ≤ 0.01). The DiD estimates indicated that the CSB+ and RUF interventions were associated with increases in mean Hb concentration of 2.25 g/dL and 2.95 g/dL, respectively, relative to the control arm (IYCF). Between the intervention arms, the RUF arm demonstrated a slightly higher increase in mean Hb concentration (DiD; 0.71 (0.36) g/dL, *p* ≤ 0.1) compared to the CSB+ arm.

**Table 3:** Difference‐in‐difference (DiD) of change in mean hemoglobin (Hb) concentration between intervention arms.

Control			Intervention			Difference in difference (DiD)
Baseline Mean (g/dL)	End line Mean (g/dL)	Diff (E–B) Mean (SE)	Baseline Mean (g/dL)	End line Mean (g/dL)	Diff (E–B) Mean (SE)	
**Standard of care**			**RUF**			
11.08	10.83	−0.25 (0.21)	9.49	12.19	2.70**^∗∗∗^** (0.28)	2.95**^∗∗∗^** (0.35)
**Standard of care**			**CSB**			
11.08	10.83	−0.25 (0.21)	9.88	11.88	2.00**^∗∗∗^** (0.22)	2.25**^∗∗∗^** (0.30)
**CSB**			**RUF**			
9.88	11.88	2.00^**∗∗∗**^(0.22)	9.49	12.19	2.70**^∗∗∗^** (0.28)	0.71**^∗^** (0.36)

B: baseline; CSB+: cereal soy blend; DiD: difference in difference; Diff: difference; E: end line; IYCF: infant and young child feeding; RUF: ready‐to‐use food supplement.

^∗∗∗^and ^∗^indicate significance at 0.01 and 0.1, respectively.

Table 4 presents the DiD analysis of the change in mean Hb concentration between the intervention arm (RUF) and the control arm, considering the characteristics of mother–child pairs. The RUF intervention was efficacious in increasing the mean Hb concentration among study participants across all characteristics of mother–child pairs, except for the caregiver’s education (*p* < 0.05), when compared to the control arm. There was no significant difference in the increases in mean Hb concentration among mother–child pairs with secondary/higher education level (*p* ≥ 0.05). In particular, within the intervention arm (RUF), there was an increase in mean Hb concentration at the end line compared to baseline across all mother–child pair characteristics. In contrast, in the control arm, there was a decrease in mean Hb concentration at the end line compared to baseline among children who were not being breastfed and those who had experienced fever within the two weeks before the survey.

**Table 4:** Difference in difference (DiD) in change in mean hemoglobin (Hb) concentration between each intervention arm and control arm against mother–child pair characteristics.

	Control			Intervention			Difference in difference (DiD) (SE)
	Baseline *N* = 90 Mean (g/dL)	End line *N* = 84 Mean (g/dL)	Diff (E–B) Mean (SE)	Baseline *N* = 91 Mean (g/dL)	End line *N* = 84 Mean (g/dL)	Diff (E–B) Mean (SE)	
	Standard of care			RUF			
**Sex of child**							
Male	11.19	10.8	−0.39 (0.28)	9.59	12.2	2.61^**∗∗∗**^ (0.41)	3.00^**∗∗∗**^ (0.50)
Female	11.00	10.86	−0.14 (0.29)	9.41	12.19	2.78^**∗∗∗**^ (0.39)	2.92^**∗∗∗**^ (0.49)
**Caregiver education**							
No formal education	10.85	10.40	−0.45 (0.40)	9.07	11.79	2.72^**∗∗∗**^ (0.51)	3.18^**∗∗∗**^ (0.64)
Primary education	11.20	11.02	−0.18 (0.25)	9.84	12.54	2.70^**∗∗∗**^ (0.35)	2.87^**∗∗∗**^ (0.43)
Secondary/higher	11.20	11	0.20 (0.45)	8.68	11.39	2.71^**∗∗∗**^ (1.01)	2.51 (1.49)
**Employment status**							
Unemployed	11.60	11.51	0.09 (0.32)	10.17	8.34	−1.83^**∗**^ (0.89)	−1.92^**∗**^ (0.74)
Employed	11.00	11.3	−0.30 (0.23)	9.46	6.71	−2.75^**∗∗∗**^ (0.3)	−2.45^**∗∗∗**^ (0.93)
**Number of children**							
1–3	11.20	10.83	−0.37 (0.35)	9.39	12.07	2.68^**∗∗∗**^ (0.41)	3.05^**∗∗∗**^ (0.55)
4–6	11.14	10.99	−0.15 (0.27)	9.83	12.32	2.49^**∗∗∗**^ (0.43)	2.64^**∗∗∗**^ (0.49)
7+	10.55	10.3	−0.25 (0.63)	8.83	12.28	3.45^**∗∗∗**^ (0.97)	3.69^**∗∗∗**^ (1.16)
**Marital status**							
Single parent	11.69	11.81	−0.12 (0.42)	9.31	6.43	2.88^**∗∗∗**^ (0.84)	3.00^**∗∗∗**^ (0.93)
Married/cohabit	10.95	11.23	−0.28 (0.23)	9.53	6.86	2.67^**∗∗∗**^ (0.3)	2.95^**∗∗∗**^ (0.38)
**Still breastfeed**							
Yes	11.16	11.11	−0.05 (0.29)	9.08	11.9	2.82^**∗∗∗**^ (0.41)	2.87^**∗∗∗**^ (0.51)
No	11.01	10.51	−0.50^**∗**^ (0.29)	10.13	12.53	2.40^**∗∗∗**^ (0.35)	2.90^**∗∗∗**^ (0.45)
**Age of the child**							
< 24 months	11.12	10.92	−0.20 (0.26)	9.11	11.87	2.76^**∗∗∗**^ (0.36)	2.96^**∗∗∗**^ (0.45)
≥ 24 months	11.00	10.7	−0.30 (0.35)	10.40	12.8	2.40^**∗∗∗**^ (0.38)	2.70^**∗∗∗**^ (0.52)
**Had a fever in the last 2 weeks**							
Yes	11.15	11.98	−0.83^**∗∗**^ (0.32)	9.53	12.11	2.58^**∗∗∗**^ (0.59)	3.41^**∗∗∗**^ (0.62)
No	11.05	11.12	0.07 (0.27)	9.47	6.72	−2.75^**∗∗∗**^ (0.33)	−2.82^**∗∗∗**^ (0.44)
**Had diarrhea in the last 2 weeks**							
Yes	11.08	11.38	−0.3 (0.42)	9.43	7.61	−1.82^**∗∗∗**^ (0.62)	−1.52^**∗∗∗**^ (0.78)
No	11.09	11.33	−0.24 (0.24)	9.54	6.72	−2.82^**∗∗∗**^ (0.34)	3.06^**∗∗∗**^ (0.41)
**Minimum dietary diversity (MDD)**							
No	11.07	11.21	−0.14 (0.21)	9.68	7.32	−2.36^**∗∗∗**^ (0.3)	−2.22^**∗∗∗**^ (0.36)

*Note:* The number of mother–child pairs who met the minimum dietary diversity score was not enough observations in each combination of treatment and time (survey period) to make the estimates. B: baseline; Diff: difference; E: end line; RUF: ready‐to‐use food supplement.

Abbreviations: DiD, difference in difference; IYCF, infant and young child feeding.

^∗∗∗^, ^∗∗^, and ^∗^indicate significance at 0.01, 0.05, and 0.1, respectively.

### 3.4. Anemia Status at End Line Before and After Interventions

Table 5 presents the changes in anemia prevalence before and after the intervention period in both the intervention and control arms. A reduction in anemia prevalence of 65.50% was observed among children in the RUF intervention arm (*p* < 0.05). However, there was a slight increase in anemia prevalence in the intervention arm overall. At the end line, anemia prevalence was higher in the control arm compared to the intervention arm (*p* < 0.001).

**Table 5:** Proportion of children with anemia between intervention arms and control at baseline and end line.

	Total *n* (%)	Standard of care *n* (%)	RUF *n* (%)	*p*‐value
Baseline	101 (60.1)	34 (40.5)	67 (79.8)	
End line	49 (29.1)	37 (44.0)	12 (14.3)	< 0.001

*p* < 0.05 indicates significance.

## 4. Discussion

Supplementation with RUF and CSB+ for children aged 6–59 months improved Hb concentration in the intervention arms compared to the control arm. The mean increase in Hb levels across all arms (RUF, CSB+, and standard of care) showed a significant difference (*p* ≤ 0.001). The RUF demonstrates a higher mean increase compared to both the CSB+ and standard of care arms. Fortifying locally produced RUF with micronutrients enhanced the likelihood of achieving higher Hb levels among infants and young children. The provision of iron through fortified foods has been shown to be an efficacious strategy in preventing and treating ID and ID anemia (IDA) in this population [16]. However, infants and young children are particularly vulnerable to IDA due to the depletion of iron stores from rapid growth and low iron content in their diets [17]. The currently recommended iron content of RUTF is 10–14 mg/100 g, aiming to address ID [18]. Both RUF and CSB+ products met the recommended iron content; thus, the difference in mean Hb increases may be attributed to individual physiological variables such as body iron status [19]. Several studies have reported an inverse relationship between body iron stores and iron absorption; more iron is absorbed in an iron‐deficient state, whereas less iron is absorbed when iron stores are sufficient [20, 21].

The trial demonstrated that supplementation with RUF was efficacious in reducing the prevalence of anemia from 79.80% at baseline to 14.30% at the end of the study. These findings are consistent with other studies that assessed the effects of RUF supplements on Hb concentration. For instance, a study reported the impact of RUF on increased mean Hb concentration by 3.80 g/L (95% CI: 0.6, 7.0; *p* = 0.02) for children in the intervention group [22]. Similarly, the study assessed the efficaciousness of locally produced ready‐to‐use supplementary food on Hb concentration among children aged 6–23 months, revealing a significant increase in Hb levels in the intervention arm compared to the control arm (*p*  <  0.001) [23]. Improved iron status and Hb concentration were also found in a study conducted in Burkina Faso that examined the effects of additional meals for the treatment of MAM on inflammation, malaria, and Hb in children with MAM [24]. Likewise, the study conducted in Dhaka demonstrated a noteworthy rise in Hb content in children aged 6–23 months who were supplemented with micronutrient powder [25].

In contrast to our findings, the study conducted in Uganda did not observe any difference (*p* = 0.115) in mean Hb levels between the treatment group (10.15 g dL^−1^) and the control group (10.46 g dL^−1^) at the end of the intervention; both the levels were observed to be below the WHO reference cutoff value of 11.00 g dL^−1^ for healthy children [26]. The increased effect of RUF on raising Hb concentration and lowering the prevalence of ID in the intervention group could be explained by a number of different mechanisms. Absorption of iron from the diet is influenced by several factors including the type of iron, presence of infections, the amount of iron reserves in the body, and the availability of iron enhancers (like vitamin C) or iron inhibitors (like phytate and polyphenols) [27]. Research has shown that approximately 53% of the vitamin C in CSB is lost during cooking; therefore, even though the products had the same type of iron, the presence of iron inhibitors or enhancers may have an impact on absorption [28]. Therefore, the reduced prevalence of anemia in RUF arms could be attributed to the convenience of RUF, which requires no cooking. This aspect may have encouraged mothers to adhere to feeding schedules, potentially contributing to the efficaciousness of the RUF product in increasing Hb levels. Additionally, the provision of RUF was accompanied by specific instructions that it should be used solely for feeding identified MAM children only and not for others. The product was portrayed as a medicinal intervention, with discouragement against sharing it with other children in the household.

Food consumption is one of the most important factors in the etiology of nutritional anemia and ID in children. Dietary diversification may help to alleviate multiple micronutrient deficiencies and reduce the risk of nutritional anemia [26]. In low‐ and middle‐income countries, including Tanzania, the diets of the majority of children lack diversity and primarily consist mainly of plant‐based foods, with limited consumption of animal source foods, fruits, and vegetables [29, 30]. The findings showed that dietary diversification was very low among recruited children, with 83.70% not meeting the recommended MDD of four or more food groups based on the WHO recommendations. This aligns with a study conducted in Tanzania, which reported that 74% of children did not attain MDD [31]. Arms with a higher proportion of children who met the MDD also experienced a significant change in Hb concentration (*p* < 0.05). The likelihood of being anemic increases as the number of food groups consumed decreases. Dietary diversity is directly related to the quality of diet and inversely related to malnutrition in terms of inadequate nutrient intake. Studies report that cereals and legumes are the major food groups most commonly consumed by children under the age of five [32, 33]. However, cereals and legumes naturally contain antinutritional factors that limit the accessibility of minerals. Individuals who consume predominantly cereal‐based diets are at a greater risk of developing ID due to the high content of iron inhibitors, such as phytic acid, in the diets [34].

Furthermore, limited consumption of animal food sources, which are an excellent source of iron, has been noted. Food items such as meat, eggs, fruits, milk, and milk products are typically consumed less frequently. Increasing the intake of animal source foods, along with fruits and vegetables rich in vitamin C and carotenoids, may enhance the absorption of iron from cereal‐based diets. In addition, the consumption of animal food sources contributes to the dietary intake of nutrients such as highly bioavailable heme iron, vitamin B_12_, and vitamin A, playing an important role in reducing the risk of developing anemia [35]. Differences in Hb response between the RUF and CSB+ arms may be partially explained by variations in iron bioavailability influenced by the presence of antinutritional and enhancing factors. In contrast, RUF formulations fortified with iron improve bioavailability. The compositional differences highlight the importance of considering both antinutritional inhibitors and absorption enhancers when evaluating the effectiveness of complementary foods in addressing anemia among children with MAM.

The main strength of this study is the use of randomized controlled trials, the gold standard for assessing the efficacy of any newly developed food product as it provides reliable and unbiased evidence of its efficaciousness. The main drawbacks of this study were recall bias and the use of a single 24‐h dietary assessment to measure dietary intake, which may not accurately reflect the children’s typical diet quality and underrepresent habitual intakes; hence, it should be interpreted with caution. Additionally, adherence data were not quantified in this study, which might weaken the reliability of the dose–effect relationship.

## 5. Conclusion

The study provides strong evidence that RUF is effective in improving Hb concentration and reducing anemia among moderately malnourished children in Tanzania. The results highlight the importance of incorporating RUF into child nutrition programs to enhance nutritional status and health outcomes. Scaling up its use could contribute to national and global efforts to combat malnutrition in vulnerable populations.

## 6. Limitation

Despite the study’s robust design, baseline imbalances in caregiver education and dietary diversity were observed between groups. These factors may have influenced household food practices and child dietary outcomes, potentially introducing bias in the estimated intervention effects. Although the overall direction of findings aligns with the study’s theoretical expectations, future research should consider using adjusted analyses or stratified sampling approaches to better control for such differences.

Reliance on a single 24‐h dietary recall may not fully capture habitual dietary intake and could lead to misclassification of diet quality. Day‐to‐day variation in consumption, seasonal influences, and recall bias may have affected the accuracy of dietary assessment. Despite this, the 24‐h recall remains a practical and widely used method in field‐based nutritional studies.

## Conflicts of Interest

The authors declare no conflicts of interest.

## Funding

This study was funded by the World Food Programme. However, no specific grant was received from any funding agency for publication.

## Data Availability

The data that support the findings of this study are available in a publicly accessible repository and can be accessed through https://figshare.com/s/be05b3c213dfd87a06fe.
